# Frequency of depression, anxiety and stress among university students

**DOI:** 10.12669/pjms.36.5.1873

**Published:** 2020

**Authors:** Saba Asif, Azka Mudassar, Talala Zainab Shahzad, Mobeen Raouf, Tehmina Pervaiz

**Affiliations:** 1 Ms. Saba Asif, Associate Lecturer, Government College Women University, Sialkot, Pakistan; 2 Azka Mudassar, Department of Psychology, Government College Women University, Sialkot, Pakistan; 3 Talala Zainab Shahzad, Department of Psychology, Government College Women University, Sialkot, Pakistan; 4 Mobeen Raouf, Department of Psychology, Government College Women University, Sialkot, Pakistan; 5 Tehmina Pervaiz, Department of Psychology, Government College Women University, Sialkot, Pakistan

**Keywords:** Anxiety, DASS-21, Depression, Stress, Prevalence, Students

## Abstract

**Objective:**

The main objective of the present study was to explore the frequency of Depression, anxiety and stress among university students in Sialkot, Pakistan.

**Method:**

Survey research method was used to collect data from three universities of Sialkot by using simple random sampling technique from 500 university students. The study was conducted at GC Women University, Sialkot in total duration of five months from February 2019 to June 2019. A demographic sheet and DASS-21 (Depression, Anxiety Stress Scale) were used to measure the level of depression, anxiety and stress. Data was scored according to the standard scoring procedure for each subscale and for further analysis frequency distribution method was applied through statistical package for social sciences (SPSS. 21).

**Results:**

The means of Depression, Anxiety and stress are M=15.08, M=18.24 and M=19.02 respectively. The frequency of depression, anxiety and stress among university students was found 75%, 88.4% and 84.4% respectively. The findings of the study showed the prevalence of Depression within the range of normal (25%), mild (16%), moderate (35.8%), severe (14.6%) and extremely severe (8.6%). The prevalence of anxiety was found to be in the range of normal (11.6%), mild (4.4%), moderate (19.4%), severe (17.8%) and extremely severe (46.8%). Stress was normal (15.6%), mild (33.8%), moderate (35.4%), severe (13.2%) and extremely severe (2.8%).

**Conclusion:**

It is concluded that symptoms of anxiety and stress are more prevalent with moderate to extremely severe range than depression in the current sample. These findings suggest urgent need of some preventive measures and interventions to improve the mental health of students.

## INTRODUCTION

Students are a unique group of people who are passing from most critical period of life in which they experience many stressful events.^1^ As the education proceed to the higher level the students use to face more stressful events like more tough syllabus, challenging work assignments and projects, residing in hostels such challenges need to be cope affectively. It is the duty of educator to help their students to cope from such stressors which allow them to have a stable mental health.^2^ Depression is a multi-problematic disorder that cause heavy burden in society which leads to the impairment of individual, social, interpersonal and occupational functioning.^3^ Anxiety is an internalized arousal of fear that may be real or imaginary. Anxiety is an unconscious reaction to depressive tendencies which may turn into severe fear or panic. Moreover, anxious students are also reported to suffer from leaning difficulties and problem solving. The psychological and physical symptoms include shivering of hands and lips, dryness in mouth, frequent urination and restless sleep.^4^ Stress is defined as a threat that poses challenge to our well-being. When an organism adaptive capacity does not work accordingly to the demands of environment, results in biological and psychological disturbances.^5^ The earlier literature on mental health problems indicated that the students are expected to be well prepared for the future demands, stressors, increased responsibilities in academic as well as social life which leads to mental health problems among university students. This prevalence and frequency are varied across the globe due to various factors. Currently, it is considered that mental health issues are a very crucial public health concern resulting into one third of disability worldwide.^6^

A study on Australian university students reported that 53% students suffered from psychological distress.^7^ In a sample of university student in Turkey were found to have Depression (27.1%), anxiety (47.1%) and stress (27%) respectively.^8^ In addition, 30% undergraduate students in Canada showed psychological issues,^9^ 41.9% medical students were found to had emotional disorders in Malaysia.^10^ Asian counties reported to suffer more from depression, anxiety and stress than the other countries. A study conducted on the medical students in India reported high level of depression, anxiety and stress 51.3%, 66.9%, 53% respectively.^11^ Earlier research evidence on DAS among university students in Pakistan suggested high level of prevalence of depression and anxiety.

A study in 2013 reported 7.5% low level of stress, 71.67% moderate level of stress and 20.83% with high level of stress among medical college of Pakistan.^12^ In a study on medical students in Wah Pakistan reported as high level of anxiety 47.7% than depression which was 35.1%.^13^ The prevalence of depression was found 53.43% among university students in Karachi, Pakistan. Females scored significantly higher level of depression 61% than male students as 38.0% in the sample.^14^ Another research conducted on medical students in Islamabad reported high level of prevalence of depression 40.9% and anxiety 74.2% in the sample.^15^ Medical students of Nishtar Medical College, Multan were found to have high level of anxiety and depression.^16^ Another study on Pakistani medical students concluded that academic stressors play a significant role to inspire learning and building sense of competition in students. But at the same time these stressors can be a reason to feel helplessness and promote anxiety which adversely affects the academic performance of students.^17^

There was no earlier evidence investigating the prevalence, frequency and severity levels of depression, anxiety and stress among university students in Sialkot city Pakistan. There are currently three universities in the Sialkot city. Therefore, an attempt was made to investigate the mental health problems among this sample.

## METHODS

The sample study was comprised of 500 students (*N=500, Girls=248 & Boys=252)* with in the age range between 18 to 24 years. The study was conducted at GC Women University, Sialkot with the ethical approval of the institute (Ref. No.: D/REG/19/2607). The total duration of this research was five months from February, 2019 to June, 2019. The simple random sampling technique was used by selecting a sample of 500 students from one public and two private universities. The data was collected with the permission of concerned authorities of relevant institutions, chosen from the city. Consent forms and demographic sheets were given to students and informed about the objective and their participation in the study. A self-developed demographic sheet was used to collect demographic information. The level of mental health was assessed by using Depression, Anxiety and Stress (DASS-21) scale (short version) among students to collect data. The scale DASS-21 consists of 21 items specifically designed to assess severity level of depression, anxiety and stress. Lovibond, S.H. Lovibond, P.F. had developed this scale in 1995 pertaining good validity and reliability.^18^ At first, universities were contacted for the permission for data collection. The sample of 500 students was selected and informed consent was signed by the participants. The participants were explained about the research purpose and its objectives. The demographic sheets, informed consents and (DASS-21) were given to participants and requested them to fill all of them. Scoring was done according to the set criteria for this scale.

The data was analyzed by a computer software SPSS-21. Mean scores of responses of the whole sample on items of (DASS-21) was computed and tabulated. Further the level of disturbance on each sub-scale (normal, mild, moderate, severe, extremely severe) was computed through frequency and percentage method.

## RESULTS

The demographic characteristics of current sample is shown in Table-I. There were 50.4% males and 49.6% female adolescents. 23.6% students were from inter, 20% from 1-2 semesters, 28.4% were from 3-4 semester, 14.6% were from 5-6 semester and 13.2% from 7-8 semester. There were 66.6% students who were in the age range of 18 years to 20 years and there were 33.4% students who were in the age range of 21 years to 24 years. 9.8% of the student’s mother were working and 90.2% were as housewives. 8.8% student’s mothers were illiterate, 14.6% had completed their middle school, 33.4% did matriculation, 18.4% had completed inter level education, 19% were graduated and 5.8% had master’s degree. 4.6% of student’s fathers were illiterate, 9.6% had completed their middle school, 31.6% did matriculation, 19.2% had completed inter level education, 21.4% were graduates and 13.6% had master’s degree.30.2% student’s fathers had their own business, 64.4% were doing job and 5.4% were jobless. 41.4% of the students were in private institutions and 58.6% were from government institutions. 99.2% of the students were Muslim and remaining 0.8% were from other religions.

**Table-I:** Demographic Characteristics of the Entire sample (N = 500).

Variables	Categories	f	%
Gender	Female	248	49.6
	Male	252	50.4
Current semester	1^st^ and 2^nd^ year	118	23.6
	1^st^ and 2^nd^ semester	101	20.0
	3^rd^ and 4^th^ semester	142	28.4
	5^th^ and 6^th^ semester	73	14.6
	7^th^ and 8^th^ semester	66	13.2
Age	18 years to 20 years	333	66.6
	21 years to 24 years	167	33.4
Mother’s profession	Working women	49	9.8
	Housewife	451	90.2
Mother’s Education	Illiterate	44	8.8
	Middle	73	14.6
	Matric	167	33.4
	Inter	92	18.4
	BA	95	19.0
	Masters	29	5.8
Father’s Education	Illiterate	23	4.6
	Below middle and middle	48	9.6
	Matric	158	31.6
	Inter	96	19.2
	BA	107	21.4
	Masters	68	13.6
Father Profession	Businessman	151	30.2
	Doing job	322	64.4
	Jobless	27	5.4
Institute category	Private	207	41.4
	Government	293	58.6
Religion	Islam	496	99.2
	Other	4	0.8

***Note: f:*** frequency, %: percentage.

The means, standard deviation and percentage scores for depression, anxiety and stress respectively is shown in Table-II. The findings suggested that overall anxiety is the most prevalent problem with 88.4%. stress= 84.4% is the second most prevalent problem and depression is 75%% in the current sample.

**Table-II:** Mean, Standard Deviation, frequency and percentage scores of DASS-21 (depression, anxiety, stress) f (%) (N=500).

Scores of DASS-21 sub-scales	Mean	Standard Deviation	f (%)
Depression	15.08	7.80	375 (75%)
Anxiety	18.24	8.39	442 (88.4%)
Stress	19.02	7.81	422 (84.4%)

**Fig.1:**
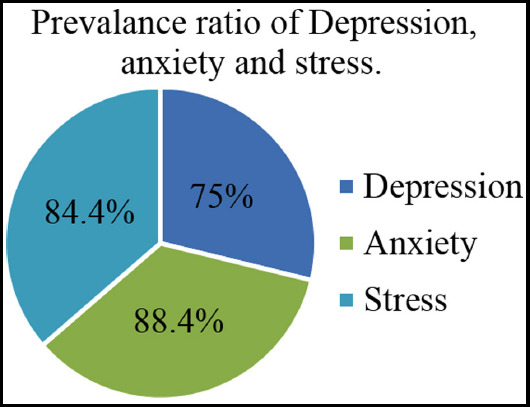
Prevalence ratio of depression anxiety and stress.

Table-III shows highest range with respect to the severity level. As for depression fall in moderate range with ratio of 35.8%, for anxiety the most prevalent range is extremely severe with ratio of 46.8% and stress fall in the moderate range with ratio of 35.4%.

**Table-III:** Frequency in weighted percentages of DASS-21 among students in Sialkot with respect to severity (Depression, anxiety and stress) (N= 500).

DASS-21	Normal	Mild	Moderate	Severe	Extremely Severe
Depression	125 (25%)	80 (16%)	179 (35.8%)	73 (14.6%)	43 (8.6%)
Anxiety	58 (11.6%)	22 (4.4%)	97 (19.4%)	89 (17.8%)	234 (46.8%)
Stress	78 (15.6%)	165 (33%)	177 (35.4%)	66 (13.2%)	14 (2.8%)

**Fig.2:**
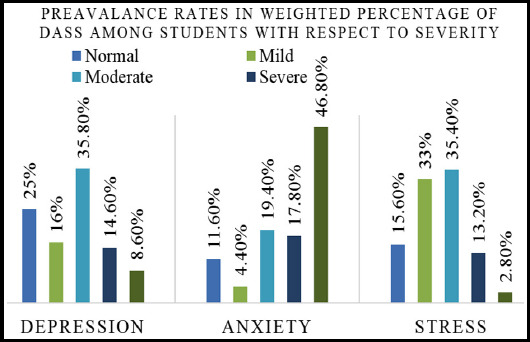
Severity level of depression, anxiety and stress. (Normal, Mild, Moderate, severe).

## DISCUSSION

The present study aimed to assess the prevalence of mental health issues (Depression, anxiety and stress) in the students. The severity level of each problem was also investigated in the current sample. The findings showed the mean of depression, anxiety and stress M=15.08, M=18.25 and M=19.03 respectively. The frequency of depression, anxiety and stress among university students was found 75%, 88.4% and 84.4% respectively in the current research. These overall frequencies were calculated by adding all the severity categories from mild, moderate, severe and very severe of each of the sub-scale as seen in Table-II. The current findings revealed that the anxiety is the most prevalent issue among the current sample with highest percentage 88.4%. The stress was the second most prevalent problem with 84.4% among university students in Sialkot. The findings suggested considerable level of frequency and severity levels of depression, anxiety and stress as psychological morbidities among the students in Sialkot, Pakistan. The prevalence of Depression with respect to severity was within the range of normal (25%), mild (16%), moderate (35.8%), severe (14.6%) and extremely severe (8.6%) in the current sample. The prevalence of anxiety was found to be in the range of normal (11.6%), mild (4.4%), moderate (19.4%), severe (17.8%) and extremely severe (46.8%). Stress was normal (15.6%), mild (33.8%), moderate (35.4%), severe (13.2%) and extremely severe (2.8%) in current findings. These findings are in line with some earlier reported findings which investigated the mental health among students in Pakistan as well as in some other countries. In contrast, the current investigation was made on the overall university students’ population within the city of Sialkot precisely 11,12,14,15.

Mental health issues containing depression, anxiety and stress is been accredited with higher level of morbidities among students around the globe. Therefore, it can be considered a subject of exploration among the researchers who are more interested in the mental health and wellbeing of the student sample. The present research has significant findings highlighting the presence of moderate to severe level psychological morbidities among the students in Sialkot, Pakistan. The response rate of participants was 100% validating the results of this study. These findings of the current study are consistent with some other studies conducted in Pakistan and in other countries. A recently reported result by a study on medical students of Karachi with 72% anxiety.^19^ Another study conducted in 2019 in Karachi on the final year of medical students reported higher level of anxiety and stress in private college students and depression was scored higher among the government college students.^20^ Another recent evidence on undergraduate students in Pakistan supports the findings of the current study. As the frequency of Depression 75%, anxiety 88.4% and stress 84.4% in current sample is greater than that 48.0% of depression, 68.54% of anxiety and 53.2% of stress respectively among the sample of undergraduate students of physiotherapy in Pakistan.^21^ There is one more research in support of the current findings which was conducted on the medical and dentistry students in Pakistan reported high prevalence of these psychological morbidities. The prevalence of anxiety was also highest with 41.9% among these students than the depression and stress.^22^

Some of the very recent evidences from other countries are also consistent with the present findings. As a study on Jordanian medical students in 2019 reported that students were suffering from psychological problems.^23^ The Prevalence of stress was found as 46.9%, anxiety was 76.2% and depression was 60.2% respectively among medical university students in Malaysia.^24^ Another research from Egypt was found consistent with the current findings reported high level of prevalence of stress 62.4%, anxiety 64.3% and depression 60.8% among university students. Anxiety was again the most prevalent problem with highest percentage of 64.3% in the sample here.^25^ A study on Indian medical students reported that half of the university students were found d to be affected by the mental health problems.^11^

### Limitations of the study

While interpreting the results, certain limitations can be kept in mind for consideration. The student’s mental health was the focus of interest and thus only depression, anxiety and stress were covered in the present investigation. The rest of the prevalent mental health problems were not examined in the current student population. Secondly, the current research only explored the frequency of these psychological morbidities, the causes of these highly prevalent issues didn’t assessed.

## CONCLUSION

It is reported by the current findings that more than half of the university students are seriously affected by mental health problems in Sialkot. It is immediately needed to design some preventive measures to promote psychological health for university students in Sialkot, Pakistan. In order to create a better learning environment and smooth functioning, it is urgently needed to focus and promote the mental health of students during their academic burden.

### Authors’ Contribution

**SA** conceived, designed, statistical analysis, revision, final approval of manuscript and is responsible for integrity of research.

**AM, TZ** review of manuscript, data collection, data entry in SPSS, manuscript writing.

**MR** did data collection, manuscript writing, editing of manuscript.

**TP** data collection, data entry, statistical analysis.
